# NFκB/Orai1 Facilitates Endoplasmic Reticulum Stress by Oxidative Stress in the Pathogenesis of Non-alcoholic Fatty Liver Disease

**DOI:** 10.3389/fcell.2019.00202

**Published:** 2019-10-02

**Authors:** Bingbing Zhang, Ming Li, Ying Zou, Han Guo, Bingdong Zhang, Cheng Xia, Hongyou Zhang, Wei Yang, Chuang Xu

**Affiliations:** ^1^College of Life Science and Technology, Heilongjiang Bayi Agricultural University, Daqing, China; ^2^College of Animal Science and Technology, Heilongjiang Bayi Agricultural University, Daqing, China; ^3^Luquan No.3 Hospital, Shijiazhuang, China

**Keywords:** NAFLD, oxidative stress, ER stress, Orai1, NEFA

## Abstract

Non-esterified fatty acids (NEFAs) promote *de novo* lipogenesis, which caused abnormal hepatic lipid accumulation, by the NFκB–Orai1 pathway. Oxidative stress and endoplasmic reticulum (ER) stress have been recognized as key mechanisms in non-alcoholic fatty liver disease (NAFLD) pathogenesis. Whether Orai1 facilitates ER stress by oxidative stress remains unknown. The rat model of NAFLD was constructed by feeding high-fat diet (HFD). BRL-3A cells were treated with NEFAs, Orai1inhibtor BTP2, NFκB inhibitor wogonin, or small interfering Orai (siOrai) 1, respectively. The content of intracellular reduced glutathione (GSH) and malondialdehyde (MDA), indicating oxidative stress, was measured by a spectrophotometer. ER stress major proteins PERK, IRE1, ATF6, CHOP, and GRP78 were quantified using Western blot and qRT-PCR analyses. For the intracellular location of reactive oxygen species (ROS) and Orai1 were measured by Western blot and immunofluorescence, and cytosolic Ca^2+^ was measured by flow cytometry. As we expected, the liver of rats with NAFLD showed lipid droplets in HE and Oil Red O. The decreased GSH and increased MDA were found in rats fed with HFD. ER stress major proteins PERK, IRE1, ATF6, GRP78, and CHOP were significantly increased in the HFD group. In BRL-3A cells, GSH content dramatically decreased from 1 h, MDA content dramatically increased from 3 h, and expression levels of ER stress significantly increased from 3 h by NEFA treatment. Furthermore, cytosolic Ca^2+^ increased from 0.5 h by NEFAs treated in BRL-3A cells. It indicated that NEFAs increased cytosolic Ca^2+^ to induce oxidative stress, thus ER stress. The content of oxidative stress and ER stress proteins showed the same trends by NEFAs treated in BRL-3A cells. These effects were reversed by the Orai1 inhibitor BTP2 and the NFκB inhibitor wogonin. Moreover, siOrai1 abrogated NEFAs’ influence in BRL-3A cells. Last, ROS was found by NEFAs treated in BRL-3A cells, and NEFA treatment enhanced the nuclear localization of NF-κB p65 and ORAI1. It was considered that high NEFAs increased cytosolic Ca^2+^ and enhanced NFκB-dependent SOCE and its moiety protein Orai1 to decrease GSH and thus induced oxidative stress at earlier stages and furthermore tempted ER stress in the pathologic progress of NAFLD.

## Introduction

Non-alcoholic fatty liver disease (NAFLD) is a common chronic health problem with increasing prevalence worldwide and progresses to non-alcoholic steatohepatitis, cirrhosis, obesity, and type II diabetes in both adults and children. The pathological characterizations of NAFLD are aberrant lipid accumulation, inflammation, and fibrotic scarring in liver. Moreover, basic and clinical evidence indicated that NAFLD has a strong relationship to insulin resistance. The two-hit model is the most widespread and prevailing theory of NAFLD. The first hit is insulin resistance, which leads to hepatic fat accumulation; the second hit is regulation of free fatty acids by the adipokines (leptin, adiponectin, resistin) to induce mitochondrial dysfunction and oxidative stress (Zeng et al., 2014). High-plasma non-esterified fatty acids (NEFAs) level is a pathological characteristic of NAFLD, obesity, and metabolic syndrome (Engin, 2017). NEFAs are any fatty acids (above C10), rather than being esterified with glycerol to form a glyceride or other lipid. Plasma NEFAs mainly include palmitic acid, palmitoleic acid, stearate, oleic acid, and linoleic acid (Diraison et al., 2003; Donnelly et al., 2005; Hetherington et al., 2016).

Previous studies have shown that NEFAs induced oxidative stress and endoplasmic reticulum (ER) stress in hepatocyte and mammary epithelial cells (Cao and Kaufman, 2014; Hasnain et al., 2016). Oxidative stress contributes to many pathological conditions, including cancer, neurological disorders, atherosclerosis, hypertension, and diabetes (Joy et al., 2017; Su et al., 2018). However, oxidative stress can be beneficial due to the attack and destruction of pathogens by the immune system (Ortiz de Orue Lucana et al., 2012). While short-term oxidative stress could prevent aging, long-term effects are caused by damage to DNA, which is induced by ionizing radiation (Czerwinska et al., 2018). Reactive oxygen species (ROS), the second signal of activating NFκB pathway, could promote the synthesis and release of inflammation cytokines in hepatocytes to induce hepatocyte inflammation response. However, to the best of our knowledge, the role of high plasma NEFAs in affecting oxidative and ER stress has not been elucidated to date.

Intracellular Ca^2+^ as the important second massager participates in cell proliferation, differentiation, migration, muscular contraction, hormone secretion, glycogen metabolism, and neuronal excitation. The homeostasis of calcium, from calcium store in ER, plays key roles in ER protein folding, mutual transport and secretion, and lipid metabolism. Previous reports indicated that insulin resistance caused Ca^2+^ concentration to increase, creating a vicious circle and promoting the pathological process of NAFLD. ER also plays key roles in reduction–oxidation balance and lipid biosynthesis. Intracellular Ca^2+^ and ROS are sensitive to protein folding in the ER. The role of cytosolic Ca^2+^ in oxidative stress and ER stress is unclear. In certain cell types, Ca^2+^ may enter through the SOCE (store-operated Ca^2+^ entry) moiety Orai1 (Prakriya et al., 2006). Notably, the expression of Orai1 has previously been shown to be regulated by nuclear factor NFκB (Lang and Shumilina, 2013). The present study addresses the question of whether Orai1 facilitates ER stress by oxidative stress in the pathogenesis of NAFLD.

## Materials and Methods

### Animal Experiments and Samples Collection

The animal experiments were performed in accordance with the Guiding Principles of Animals adopted by the Chinese Association for Laboratory Animal Sciences for the welfare of animals. The present study protocol was approved by the Ethics Committee for the Use and Care of Animals, Heilongjiang Bayi Agricultural University (Daqing, China). For our study, 18 rats 6–8 weeks of age had free access to food and tap water, and randomly divided into two groups. The nine rats of the high-fat diet (HFD) group were fed a HFD containing 70% kcal from fat (Altromin, Shanghai, China) for 8 weeks to induce NAFLD. The nine rats of the control group were fed normal chow. All animals had free access to food and tap water. Part of the liver was collected and immediately shock-frozen in liquid nitrogen and lysed in lysis buffer for Western blots, GSH, and MDA measurements, and another for H&E and Oil Red staining. The blood was collected from postcaval vein to measure non-esterified free fatty acids through the Non-esterified Free fatty acids kit (Beijing Jiuqiang Biotechnology Co., Ltd., Beijing, China) with an automatic clinical analyzer (Synchron DXC800; Beckman Coulter, Inc., Brea, CA, United States).

### Cell Culture

BRL-3A rat liver cells were obtained from ATCC and cultured in DMEM high-glucose medium (Gibco, Shanghai, China) supplemented with 10% FBS (Clark, Australia) and 1% penicillin (100 U/mL)/streptomycin (100 μg/mL) liquid (Solarbio, Beijing, China) at a 37°C incubator with 5% CO_2_. Cells were treated with 1.2 mM NEFAs for different time durations or treated with vehicle only.

Cells were pretreated with 20 μM CRAC Channel Inhibitor BTP2 (Sigma, Shanghai, China) and 100 μM NFκB inhibitor wogonin (Sigma) or treated with vehicle only. After 12 h, cells were in addition treated with 1.2 mM NEFAs for 12 h.

### Prepared NEFAs

The composition and concentration of NEFAs used in this study were according to previous descriptions (Li et al., 2013; Abdelmagid et al., 2015; Plos One Staff, 2015; Zhang et al., 2018).

### Silencing

For silencing, 3 × 10^5^ cells (6-well plate) were seeded and incubated in antibiotic-free medium for 24 h before silencing treatment. Afterward, cells were transfected with 40 pM rat Orai1 siRNA (GenePharma, Shanghai, China) and non-targeting siRNA (GenePharma, Shanghai, China) using Lipofectamine 2000 Transfection Reagent (Invitrogen, Shanghai, China) according to the manufacturer’s protocol^1^.

### Quantification of mRNA Expression

Total RNA was extracted from BRL-3A cells of the above treatments with Trizol RNA extraction reagent (Invitrogen Corporation, Carlsbad, CA, United States); mRNA transcription and cDNA synthesis were performed with Reverse Transcriptase M-MLV (RNase H-) (Takara Bio, Beijing, China) using an oligodT18 primer. Quantitative RT-PCR was performed by FastStart Universal SYBR Green Master (F. Hoffmann-La Roche AG, Basel, Switzerland) under the following conditions: 95°C for 3 min, followed by 40 cycles at 95°C for 10 s and 58°C for 30 s on a BioRad iCycler iQTM Real-Time PCR Detection System (Bio-Rad Laboratories Inc., Hercules, CA, United States). Calculated rat Orai1 and Grp78 mRNA expression were quantified with the 2^–ΔΔCT^ method and normalized to TATA box-binding protein (Tbp).

The following primers were used:

*Rat Tbp* (TATA box-binding protein):

forward (5′–3′): ACTCCTGCCACACCAGCC

reverse (5′–3′): GGTCAAGTTTACAGCCAAGATTCA

Rat Orai1

forward (5′–3′): CGTCCACAACCTCAACTCC

reverse (5′–3′): AACTGTCGGTCCGTCTTAT

Rat Grp78

forward (5′–3′): AACCCAGATGAGGCTGTAGCATA

reverse (5′–3′): CACAGTGTTCCTCGGAATCAGTT

### Western Blotting

PERK, IRE1, ATF6, GRP78, CHOP, Orai1, NF-κB p65, and β-actin protein levels were determined in rat livers and BRL-3A cells. The samples with RIPA lysis buffer (Beyotime Biotechnology, Shanghai, China) were incubated on ice for 30 min and later centrifuged at 14,000 rpm for 20 min (4°C). Total protein was collected and separated by SDS-PAGE and subsequently transferred to PVDF membranes and blocked in 5% non-fat milk/Tris-buffered saline/Tween-20 (TBST) at room temperature for 1 h. Membranes were incubated overnight at 4°C with polyclonal rabbit anti-PERK antibody (1:1000 in 5% BSA in TBST, Cell signaling), rabbit anti-IRE1 antibody (1:1000 in 5% BSA in TBST, abcam), rabbit anti-ATF6 antibody (1:1000 in 5% BSA in TBST, abcam), mouse anti-GRP78 antibody (1:250 in 5% BSA in TBST, Santa Cruz Biotechnology), mouse anti-CHOP antibody (1:1000 in 5% BSA in TBST, Cell signaling), mouse anti-Orai1 antibody (1:1000 in 5% BSA in TBST, Cell signaling), and mouse anti-NF-κB p65 antibody (1:1000 in 5% BSA in TBST, Cell signaling). After incubation with HRP-labeled goat anti-rabbit/mice secondary antibody (3:5000, Beyotime Biotechnology) for 1 h at room temperature, the bands were visualized with enhanced chemiluminescence reagents (Beyotime). Densitometric analysis was performed using ImageJ software. The protein expressions were quantified and normalized to β-actin antibody (3:5000, Cell signaling).

### Measurement of Intracellular Micro-Reduced Glutathione (GSH) and Malondialdehyde (MDA)

The contents of intracellular GSH and MDA were determined by a micro-reduced GSH test kit (Nanjing Jiancheng, China) and an MDA assay kit (Beyotime Biotechnology), respectively. Briefly, the intracellular reduced GSH and MDA were measured by a spectrophotometer (Synergy neo HTS multi-mode microplate reader, BioTek) at 405 or 532 nm according to the manufacturer’s instructions^2^^,^^3^.

### Measurement of ROS

A ROS assay kit (Beyotime) was used to determine the intracellular change in ROS generation in BRL-3A cells. A total of 1 × 10^5^ cell samples were incubated with 10 μM carboxy-2′,7′-dichloro-dihydro-fluorescein diacetate (DCFHDA) probe to detect intracellular ROS in PBS for 15 min at 37°C. Fluorescence was measured at 488 nm (excitation) and 525 nm (emission) by a confocal laser-scanning microscope (Leica TCS SP8; Leica, Wetzlar, Germany) with 40 × /1.3 NA differential interference contrast.

### Immunofluorescence

BRL-3A cells treated with 1.2 mM NEFAs and siOrai1 were cultured on the bottom well (Cellvis, Hangzhou, China) for 24 h and then fixed with 4% paraformaldehyde for 30 min at room temperature. BRL-3A cells were incubated with 3% Albumin Bovine V (Biosharp, Hefei, China), 5% normal goat serum (Boster, Wuhan, China), and 0.5% Triton in PBS (Biofroxx, Guangzhou, China) for 30 min at room temperature for blocking unspecific bindings. Then, rabbit anti-Orai1 and mice anti-NFκB p65 were incubated in cells overnight at 4°C in a humidified chamber. The cells were incubated with Cy3 goat anti-rabbit IgG (1:500, Beyotime) and goat anti-mice IgG-FITC (Santa Cruz Biotechnology) for 1 h at room temperature, respectively, after rinsing four times with PBS. The nuclei were stained with Hoechst 33342 dye (Beyotime) for 30 min at room temperature. Fluorescence and images were obtained with a confocal laser-scanning microscope (Leica TCS SP8).

### Statistics

Data are presented as the means ± standard error of the mean (SEM) with *n* representing the number of independent experiments. All data were tested for significance with the unpaired Student’s *t*-test or one-way analysis of variance (ANOVA) followed by a Tukey *post hoc* test. A probability (*p*) value of <0.05 was considered statistically significant.

## Results

### Feeding HFD Increased Plasma NEFA Concentration to Induce NAFLD

**FIGURE 1:**
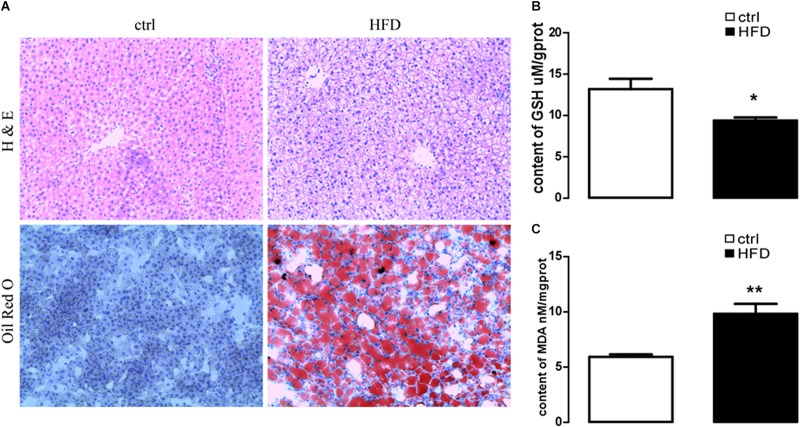
HE staining to liver steatosis and oil Red O staining to visualize lipid droplets of hepatic sections and content of GSH and MDA in rat with HFD-induced NAFLD. **(A)** HE staining (upper images) to liver steatosis and oil Red O staining (lower images) to visualize lipid droplets of hepatic sections in rats treated with HFD (right images) or normal diet (left images). **(B)** Arithmetic means ± SEM (*n* = 9) of the content of GSH in rats treated with HFD (black bar) or normal diet (white bar). **(C)** Arithmetic means ± SEM (*n* = 9) of the content of MDA in rats treated with HFD (black bar) or normal diet (white bar). ^∗^*p* < 0.05, ^∗∗^*p* < 0.01 indicate significant difference (*t*-test).

First, we addressed HFD-induced NAFLD through feeding HFD and normal diet to wild-type rats. A rat model of NAFLD was constructed according to the histopathological data. As illustrated in Figure 1A, severe panlobular micro- and macrovesicular steatosis were shown in the liver of the HFD group by H&E staining, and lipid drops were shown by Oil Red O staining. However, the liver of the control group showed normal hepatocytic texture with a large spherical nucleus and a homogeneous cytoplasm.

**FIGURE 2:**
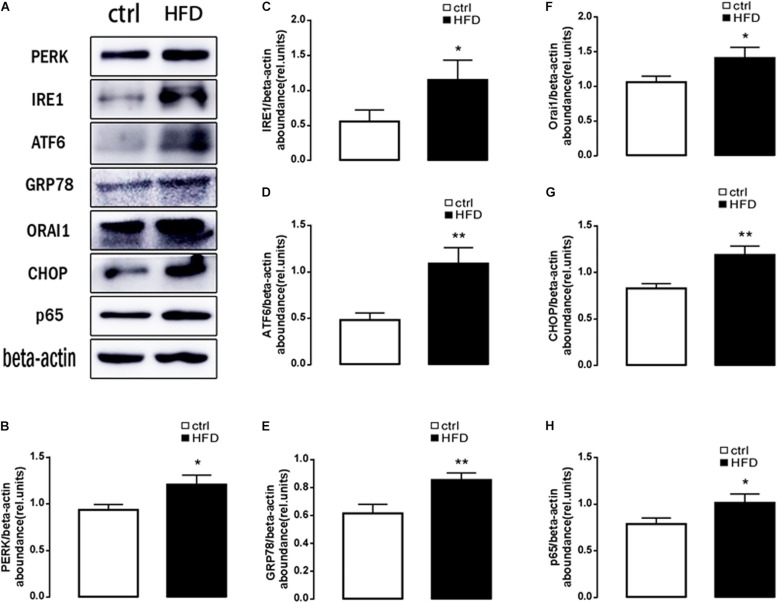
Protein expressions of ER stress, ORAI1, and NFκB p65 in rats. **(A)** Original Western blots showing the protein expression of PERK, IRE1, ATF6, CHOP, GRP78, ORAI1, NFκB p65, and β-actin in rats treated with HFD (right images) or normal diet (left images). **(B)** Arithmetic means ± SEM (*n* = 9) of the PERK/β-actin ratios in rats treated with HFD (black bar) or normal diet (white bar). **(C)** Arithmetic means ± SEM (*n* = 9) of the IRE1/β-actin ratios in rats treated with HFD (black bar) or normal diet (white bar). **(D)** Arithmetic means ± SEM (*n* = 9) of the ATF6/β-actin ratios in rats treated with HFD (black bar) or normal diet (white bar). **(E)** Arithmetic means ± SEM (*n* = 9) of the GRP78/β-actin ratios in rats treated with HFD (black bar) or normal diet (white bar). **(F)** Arithmetic means ± SEM (*n* = 9) of the ORAI1/β-actin ratios in rats treated with HFD (black bar) or normal diet (white bar). **(G)** Arithmetic means ± SEM (*n* = 9) of the CHOP/β-actin ratios in rats treated with HFD (black bar) or normal diet (white bar). **(H)** Arithmetic means ± SEM (*n* = 9) of the NFκB p65/β-actin ratios in rats treated with HFD (black bar) or normal diet (white bar). ^∗^*p* < 0.05, ^∗∗^*p* < 0.01 indicate significant difference (*t*-test).

Next, plasma NEFA was measured by Non-esterified Free fatty acids assay kit, and the results showed significantly higher (*p* = 0.0073) concentration in the HFD group (1.383 ± 0.042) than the control group (1.003 ± 0.061). Furthermore, GSH and MDA were measured by a spectrophotometer; protein expression of GRP78, PERK, IRE, ATF6, CHOP, Orai1, and NFκB p65 were measured by Western blot. As expected, the content of GSH was significantly decreased (Figure 1B); the content of MDA was increased in the HFD group (Figure 1C). PERK, IRE, ATF6, CHOP, GRP78, Orai1, and NFκB p65 proteins were also dramatically increased in the HFD group compared with the control group (Figure 2). This result indicated that in the progress of NAFLD, plasma NEFA was associated with oxidative stress and ER stress.

### High-Concentration NEFAs Induced Oxidative Stress and ER Stress in BRL-3A Cells

**FIGURE 3:**
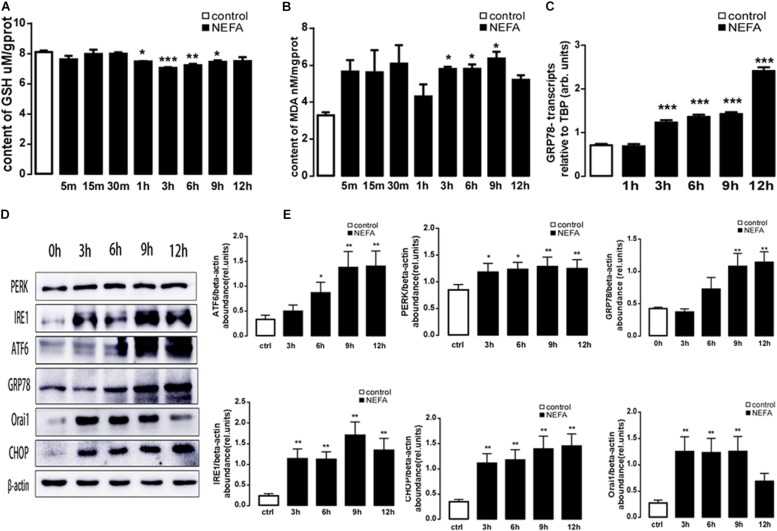
Effect of different treatment times of NEFAs on oxidative and ER stress in BRL-3A cells. **(A)** Arithmetic means ± SEM (*n* = 15) of the content of GSH in BRL-3A cells incubated without (white bar) or with (black bar) NEFAs (1.2 mM) in different times. **(B)** Arithmetic means ± SEM (*n* = 15) of the content of MDA in BRL-3A cells incubated without (white bar) or with (black bar) NEFAs (1.2 mM) in different times. **(C)** Arithmetic means ± SEM (*n* = 15) of *grp78* mRNA abundance (relative to Tbp mRNA) in BRL-3A cells incubated without (white bar) or with (black bar) NEFAs (1.2 mM) in different times. **(D)** Original Western blots showing the protein expression of PERK, IRE1, ATF6, CHOP, GRP78, ORAI1, NFκB p65, and β-actin in BRL-3A cells incubated without (white bar) or with (black bar) NEFAs (1.2 mM) in different times. **(E)** Arithmetic means ± SEM (*n* = 9) of the proteins/β-actin ratios in BRL-3A cells incubated without (white bar) or with (black bar) NEFAs (1.2 mM) in different times. ^∗^*p* < 0.05, ^∗∗^*p* < 0.01, ^∗∗∗^*p* < 0.001 indicate significant difference (one-way ANOVA).

To explore whether a high amount of NEFAs induced oxidative stress and ER stress, in the present study, BRL-3A cells were treated with high-concentration NEFAs to construct a NAFLD cell model (Zhang et al., 2018) to measure oxidative stress and ER stress. Initially, BRL-3A cells were treated with 1.2 mM NEFAs for 5 min, 15 min, 30 min, 1 h, 3 h, 6 h, 9 h, and 12 h to measure the content of MDA and GSH, and expression of GRP78, PERK, IRE, ATF6, and CHOP. As shown in Figure 3, a significant decrease in GSH level was detected from 1 to 9 h (Figure 3A), and content of MDA was significantly increased from 3 to 9 h (Figure 3B). Nevertheless, GRP78 transcription was dramatically increased from 3 to 12 h (Figure 3C), and the major ER stress-related proteins levels of PERK, IRE1, ATF6, GRP78, and CHOP were significantly up-regulated from 6 to 12 h (Figures 3D,E). These findings demonstrated that high NEFAs could facilitate the ER stress-induced subsequent reactions by oxidative stress.

### Effect of High-Concentration NEFAs on SOCE Moiety Orai1 in BRL-3A Cells

**FIGURE 4:**
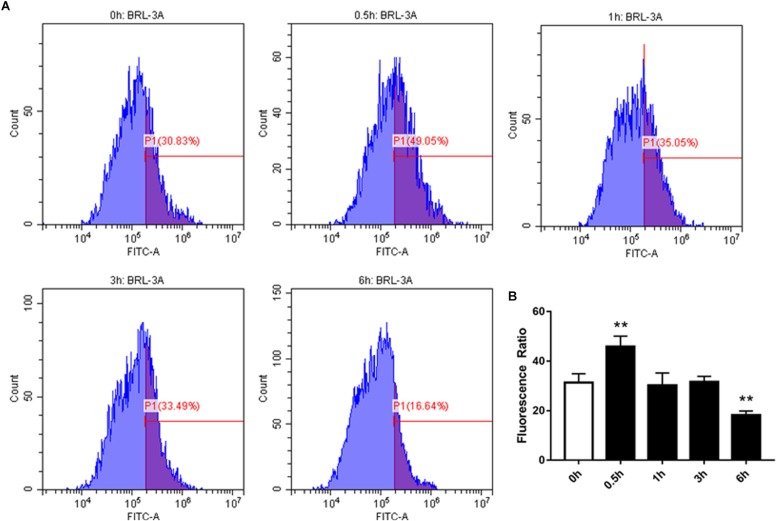
Effect of cytosolic Ca^2+^ in BRL-3A cells in different times. **(A)** Original histogram overlays of Fluo-3 fluorescence reflecting cytosolic Ca^2+^ activity in neutrophils in different times. **(B)** Arithmetic means ± SEM (*n* = 20) of Fluo-3 fluorescence reflecting cytosolic Ca^2+^ activity in neutrophils in 0 h (white bar) and different times (black bar). ^∗^*p* < 0.05, ^∗∗^*p* < 0.01 indicate difference from control (*t*-test).

We examined the effect of high-concentration NEFAs on intracellular Ca^2+^ in BRL-3A cells by Fura-3. As illustrated in Figure 4, the intracellular Ca^2+^ was found to be increased from 0.5 h in BRL-3A cells compared to the control group (Figures 4A,B). Afterward, Orai1 transcription and protein levels were increased in 3 h (Figure 3D).

**FIGURE 5:**
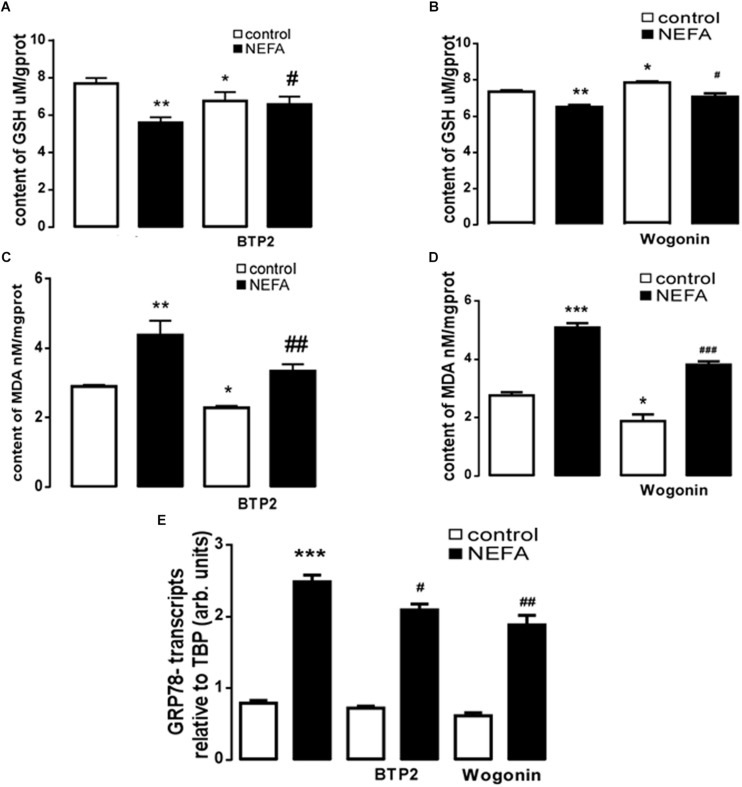
Effect of NEFAs on content of MDA and GSH, transcription of GRP78 in BRL-3A cells with or without the presence of 2-APB and Wogonin. **(A)** Arithmetic means ± SEM (*n* = 15) of the content of GSH in BRL-3A cells incubated without (white bar) or with (black bar) NEFAs (1.2 mM, 3 h) in the absence (left bars) or presence (right bars) of Orai inhibitor BTP2 (200 nM). **(B)** Arithmetic means ± SEM (*n* = 15) of the content of GSH in BRL-3A cells incubated without (white bar) or with (black bar) NEFAs (1.2 mM, 3 h) in the absence (left bars) or presence (right bars) of NFκB inhibitor wogonin (100 μM). **(C)** Arithmetic means ± SEM (*n* = 15) of the content of MDA in BRL-3A cells incubated without (white bar) or with (black bar) NEFAs (1.2 mM, 3 h) in the absence (left bars) or presence (right bars) of Orai inhibitor BTP2 (200 nM). **(D)** Arithmetic means ± SEM (*n* = 15) of the content of MDA in BRL-3A cells incubated without (white bar) or with (black bar) NEFAs (1.2 mM, 3 h) in the absence (left bars) or presence (right bars) of NFκB inhibitor wogonin (100 μM). **(E)** Arithmetic means ± SEM (*n* = 11) of Grp78 mRNA abundance (relative to Tbp mRNA) in BRL-3A cells incubated without (white bar) or with (black bar) NEFAs (1.2 mM, 12 h) in the absence (left bars) or presence (right bars) of Orai inhibitor BTP2 (200 nM) or of NFκB inhibitor wogonin (100 μM). ^∗^*p* < 0.05, ^∗∗^*p* < 0.01, ^∗∗∗^*p* < 0.001 indicate significant difference from control; #*p* < 0.05, ##*p* < 0.01, ###*p* < 0.001 indicate significant difference from NEFAs alone (one-way ANOVA).

**FIGURE 6:**
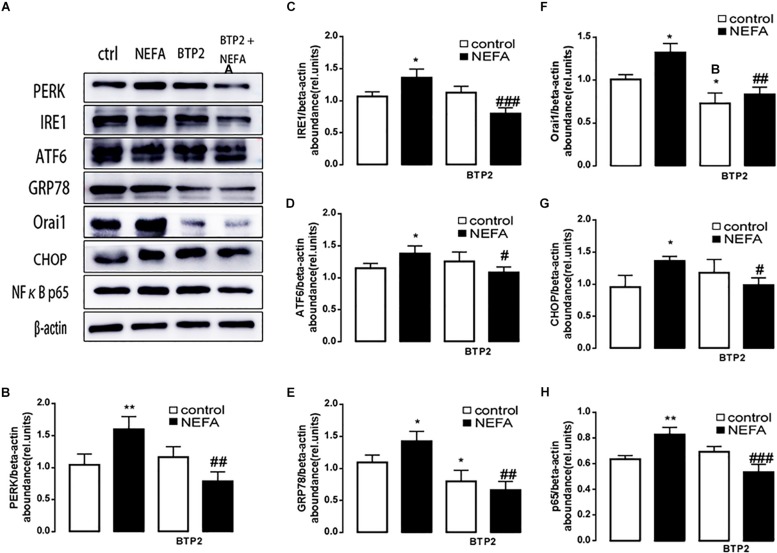
Effect of Orai inhibitor BTP2 on ER stress in BRL-3A cells. **(A)** Original Western blots showing the protein expression of PERK, IRE1, ATF6, CHOP, GRP78, ORAI1, NFκB p65, and β-actin in BRL-3A cells incubated without or with NEFA (1.2 mM, 12 h) in the absence or presence of Orai inhibitor BTP2 (200 nM). **(B)** Arithmetic means ± SEM (*n* = 9) of the PERK/β-actin ratio in BRL-3A cells incubated without (white bar) or with (black bar) NEFA (1.2 mM, 12 h) in the absence (left bars) or presence (right bars) of Orai inhibitor BTP2 (200 nM). **(C)** Arithmetic means ± SEM (*n* = 9) of the IRE1/β-actin ratio in BRL-3A cells incubated without (white bar) or with (black bar) NEFA (1.2 mM, 12 h) in the absence (left bars) or presence (right bars) of Orai inhibitor BTP2 (200 nM). **(D)** Arithmetic means ± SEM (*n* = 9) of the ATF6/β-actin ratio in BRL-3A cells incubated without (white bar) or with (black bar) NEFA (1.2 mM, 12 h) in the absence (left bars) or presence (right bars) of Orai inhibitor BTP2 (200 nM). **(E)** Arithmetic means ± SEM (*n* = 9) of the GRP78/β-actin ratio in BRL-3A cells incubated without (white bar) or with (black bar) NEFA (1.2 mM, 12 h) in the absence (left bars) or presence (right bars) of Orai inhibitor BTP2 (200 nM). **(F)** Arithmetic means ± SEM (*n* = 9) of the ORAI1/β-actin ratio in BRL-3A cells incubated without (white bar) or with (black bar) NEFA (1.2 mM, 12 h) in the absence (left bars) or presence (right bars) of Orai inhibitor BTP2 (200 nM). **(G)** Arithmetic means ± SEM (*n* = 9) of the CHOP/β-actin ratio in BRL-3A cells incubated without (white bar) or with (black bar) NEFA (1.2 mM, 12 h) in the absence (left bars) or presence (right bars) of Orai inhibitor BTP2 (200 nM). **(H)** Arithmetic means ± SEM (*n* = 9) of the NFκB p65/β-actin ratio in BRL-3A cells incubated without (white bar) or with (black bar) NEFA (1.2 mM, 12 h) in the absence (left bars) or presence (right bars) of Orai inhibitor BTP2 (200 nM). ^∗^*p* < 0.05, ^∗∗^*p* < 0.01 indicate significant difference from control; #*p* < 0.05, ##*p* < 0.01, ###*p* < 0.001 indicate significant difference from NEFAs alone (one-way ANOVA).

**FIGURE 7:**
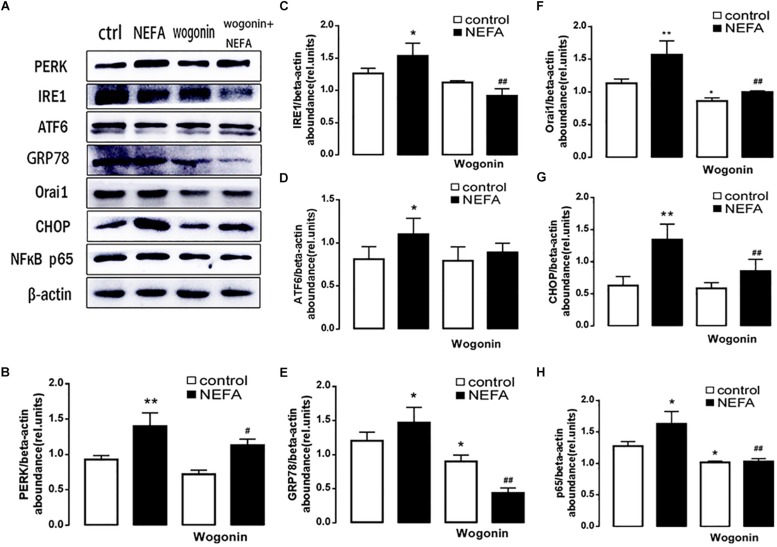
Effect of NFκB inhibitor wogonin on ER stress in BRL-3A cells. **(A)** Original Western blots showing the protein expression of PERK, IRE1, ATF6, CHOP, GRP78, ORAI1, NFκB p65, and β-actin in BRL-3A cells incubated without or with NEFA (1.2 mM, 12 h) in the absence or presence of NFκB inhibitor wogonin (100 μM). **(B)** Arithmetic means ± SEM (*n* = 9) of the PERK/β-actin ratio in BRL-3A cells incubated without (white bar) or with (black bar) NEFA (1.2 mM, 12 h) in the absence (left bars) or presence (right bars) of NFκB inhibitor wogonin (100 μM). **(C)** Arithmetic means ± SEM (*n* = 9) of the IRE1/β-actin ratio in BRL-3A cells incubated without (white bar) or with (black bar) NEFA (1.2 mM, 12 h) in the absence (left bars) or presence (right bars) of NFκB inhibitor wogonin (100 μM). **(D)** Arithmetic means ± SEM (*n* = 9) of the ATF6/β-actin ratio in BRL-3A cells incubated without (white bar) or with (black bar) NEFA (1.2 mM, 12 h) in the absence (left bars) or presence (right bars) of NFκB inhibitor wogonin (100 μM). **(E)** Arithmetic means ± SEM (*n* = 9) of the GRP78/β-actin ratio in BRL-3A cells incubated without (white bar) or with (black bar) NEFA (1.2 mM, 12 h) in the absence (left bars) or presence (right bars) of NFκB inhibitor wogonin (100 μM). **(F)** Arithmetic means ± SEM (*n* = 9) of the ORAI1/β-actin ratio in BRL-3A cells incubated without (white bar) or with (black bar) NEFA (1.2 mM, 12 h) in the absence (left bars) or presence (right bars) of NFκB inhibitor wogonin (100 μM). **(G)** Arithmetic means ± SEM (*n* = 9) of the CHOP/β-actin ratio in BRL-3A cells incubated without (white bar) or with (black bar) NEFA (1.2 mM, 12 h) in the absence (left bars) or presence (right bars) of NFκB inhibitor wogonin (100 μM). **(H)** Arithmetic means ± SEM (*n* = 9) of the NFκB p65/β-actin ratio in BRL-3A cells incubated without (white bar) or with (black bar) NEFA (1.2 mM, 12 h) in the absence (left bars) or presence (right bars) of NFκB inhibitor wogonin (100 μM). ^∗^*p* < 0.05, ^∗∗^*p* < 0.01, ^∗∗∗^*p* < 0.001 indicate significant difference from control; #*p* < 0.05, ##*p* < 0.01 indicate significant difference from NEFAs alone (one-way ANOVA).

In order to further explore whether high NEFAs facilitated ER stress by oxidative stress through the NFκB/Orai1 signaling pathway in BRL-3A cells, CRAC channel inhibitor BTP2 and NFκB inhibitor wogonin were used to detect the effect. We observed that GSH content was dramatically lower in the NEFA group than the control group (Figures 5A,B), and MDA was dramatically higher (Figures 5C,D). Conversely, the effects were abrogated by the BTP2 and wogonin, respectively. GRP78 mRNA level was significantly increased in the NEFA group; the effects were abrogated by BTP2 and wogonin (Figure 5E). The major ER stress-related proteins GRP78, PERK, IRE, ATF6, and CHOP, and NFκB p65 and ORAI1 levels were significantly increased by NEFAs and BTP2 (Figures 6A–H and Supplementary Figure S1A), and wogonin (Figures 7A–H and Supplementary Figure S1B) blocked the effects of NEFA treatment.

The final series of experiments explored whether the SOCE moiety Orai1 participates in the signaling that regulates ER stress by oxidative stress. BRL-3A cells were treated with siOrai1. To verify silencing efficiency, the transcript level and protein expression of ORAI1 were quantified. As a result, the mRNA level was 0.022 ± 0.002 a.u. (*n* = 9) in cells transfected with siOrai1 and 0.069 ± 0.011 a.u. (*n* = 9), *p* < 0.01, in cells transfected with negative control siRNA.

**FIGURE 8:**
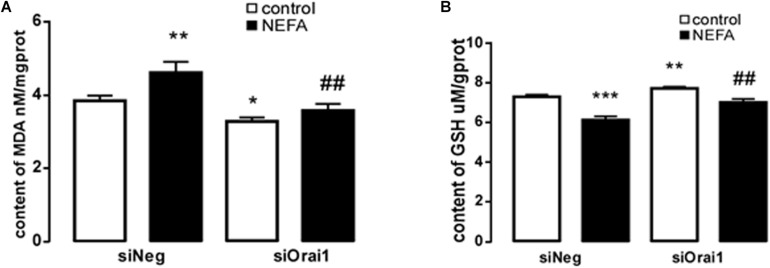
Effect of Orai1 silencing on oxidative stress in BRL-3A cells. **(A)** Arithmetic means ± SEM (*n* = 9) of the content of MDA in BRL-3A cells incubated without (white bar) or with (black bar) NEFA (1.2 mM, 12 h) in the absence (left bars) or presence (right bars) of siOrai1. **(B)** Arithmetic means SEM (*n* = 15) of the content of GSH in BRL-3A cells incubated without (white bar) or with (black bar) NEFA (1.2 mM, 12 h) in the absence (left bars) or presence (right bars) of siOrai1. ^∗^*p* < 0.05, ^∗∗^*p* < 0.01, ^∗∗∗^*p* < 0.001 indicate significant difference from control; ^#^*p* < 0.05, ^##^*p* < 0.01, indicate significant difference from NEFAs alone (one-way ANOVA).

**FIGURE 9:**
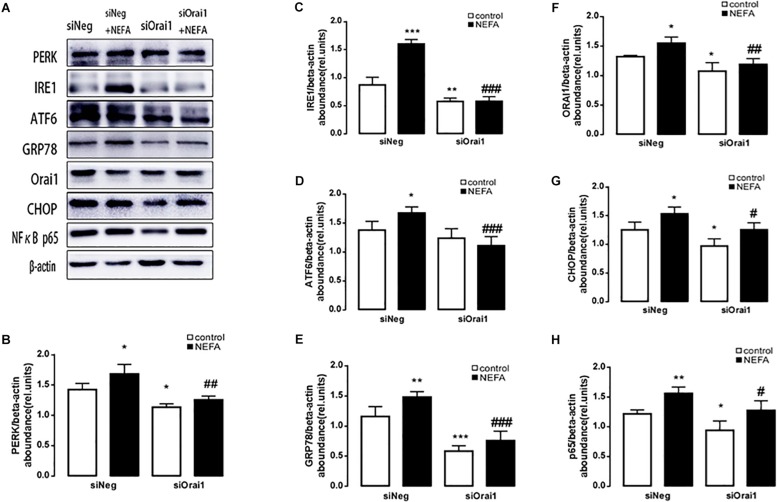
Effect of Orai1 silencing on ER stress protein levels in BRL-3A cells. **(A)** Original Western blots showing the protein expression of PERK, IRE1, ATF6, CHOP, GRP78, ORAI1, NFκB p65, and β-actin in BRL-3A cells incubated without or with NEFA (1.2 mM, 12 h) in the absence or presence of siOrai1. **(B)** Arithmetic means ± SEM (*n* = 9) of the PERK/β-actin ratio in BRL-3A cells incubated without (white bar) or with (black bar) NEFA (1.2 mM, 12 h) in the absence (left bars) or presence (right bars) of siOrai1. **(C)** Arithmetic means ± SEM (*n* = 9) of the IRE1/β-actin ratio in BRL-3A cells incubated without (white bar) or with (black bar) NEFA (1.2 mM, 12 h) in the absence (left bars) or presence (right bars) of siOrai1. **(D)** Arithmetic means ± SEM (*n* = 9) of the ATF6/β-actin ratio in BRL-3A cells incubated without (white bar) or with (black bar) NEFA (1.2 mM, 12 h) in the absence (left bars) or presence (right bars) of siOrai1. **(E)** Arithmetic means ± SEM (*n* = 9) of the GRP78/β-actin ratio in BRL-3A cells incubated without (white bar) or with (black bar) NEFA (1.2 mM, 12 h) in the absence (left bars) or presence (right bars) of siOrai1. **(F)** Arithmetic means ± SEM (*n* = 9) of the ORAI1/β-actin ratio in BRL-3A cells incubated without (white bar) or with (black bar) NEFA (1.2 mM, 12 h) in the absence (left bars) or presence (right bars) of siOrai1. **(G)** Arithmetic means ± SEM (*n* = 9) of the CHOP/β-actin ratio in BRL-3A cells incubated without (white bar) or with (black bar) NEFA (1.2 mM, 12 h) in the absence (left bars) or presence (right bars) of siOrai1. **(H)** Arithmetic means ± SEM (*n* = 9) of the NFκB p65/β-actin ratio in BRL-3A cells incubated without (white bar) or with (black bar) NEFA (1.2 mM, 12 h) in the absence (left bars) or presence (right bars) of siOrai1. ^∗^*p* < 0.05, ^∗∗^*p* < 0.01, ^∗∗∗^*p* < 0.001 indicate significant difference from control; #*p* < 0.05, ##*p* < 0.01, ###*p* < 0.001 indicate significant difference from NEFAs alone (one-way ANOVA).

**FIGURE 10:**
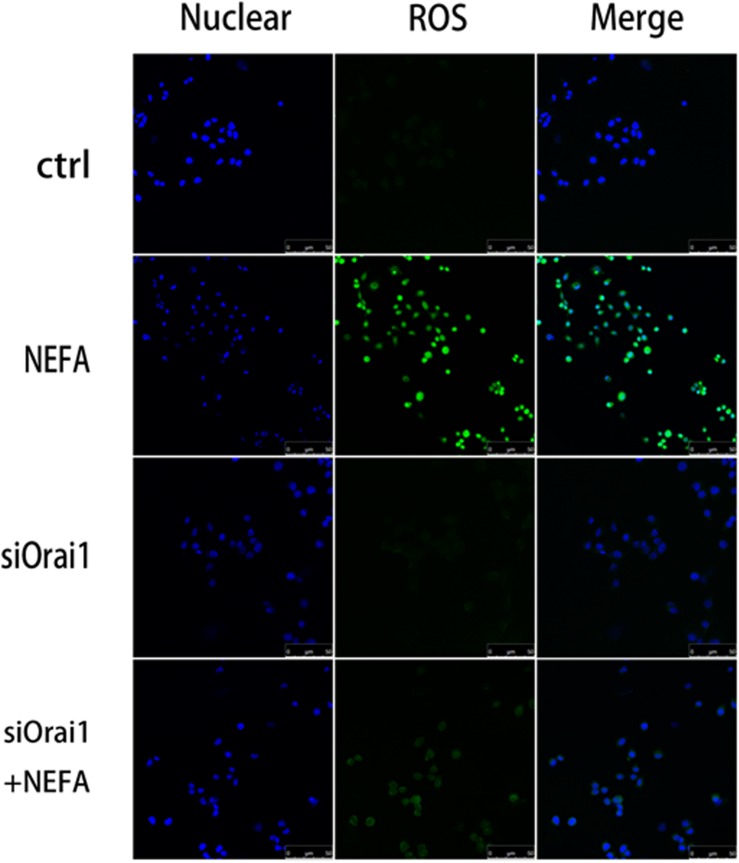
Effect of high-concentration NEFAs and siOrai1 on ROS in BRL-3A cells. Original immunofluorescence images demonstrating nuclear staining (blue; left images), ROS (green; middle images), and an overlaying of both nuclear staining and ROS (right images) in BRL-3A cells incubated without (upper images) or with (second images) NEFAs (1.2 mM, 3 h), siOrai1 (third images), or siOrai1 + NEFAs (lower images). Scale bar: 25 μm.

**FIGURE 11:**
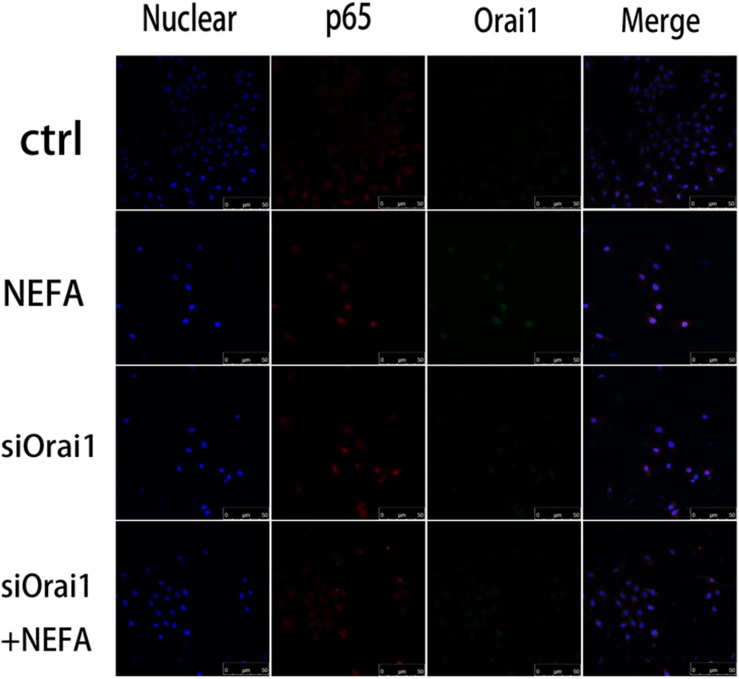
Effect of high-concentration NEFAs and siOrai1 on Orai1 and NFκB p65 localization in BRL-3A cells. Original immunofluorescence images demonstrating nuclear staining (blue; left images), NFκB p65 (red; second images), Orai1-specific antibody (Green; third images), and an overlaying of all nuclear staining NFκB p65, and Orai1-specific antibody in BRL-3A cells incubated without (upper images) or with (second images) NEFAs (1.2 mM, 3 h), siOrai1 (third images), or siOrai1 + NEFAs (lower images). Scale bar: 50 μm.

As illustrated in Figures 8A,B, GSH content was increased by siOrai1 and MDA content was decreased by siOrai1. As expected, the protein expression of GRP78, PERK, IRE, ATF6”, and CHOP, and NFκB p65 and ORAI1 levels were dramatically decreased by siOrai1 treatment (Figures 9A–H and Supplementary Figure S1C). Furthermore, as illustrated in Figure 10, ROS was increased by NEFA and reduced by siOrai1. Immunofluorescence confirmed activation of Orai1, NFκB p65, and phosph-NFκB p65 by NEFAs and siOrai1 in BRL-3A cells (Figure 11 and Supplementary Figure S2). These findings indicate that store-operated Ca^2+^ entry participated in NEFA-induced oxidative stress and ER stress.

## Discussion

The present observations reveal an important role of the store-operated Ca^2+^ entry moiety Orai1 in the regulation of ER stress by oxidative stress in the progress of NAFLD. Furthermore, in the pathologic process of NAFLD, NEFAs facilitated ER stress by oxidative stress through NFκB-dependent Orai1 signaling in BRL-3A cells.

Oxidative stress and ER stress are highly interrelated with multiple hepatic dysfunctions, including insulin resistance, lipotoxicity, inflammation, and steatosis. Mitochondrial ROS generation plays a key role in these two hits of the NAFLD process. MDA is also a marker for oxidative stress, produced by lipid peroxidation of polyunsaturated fatty acids by ROS degradation. As a reactive aldehyde, MDA is an important reactive electrophile species that could induce toxic stress in cells and form advanced lipoxidation end products (ALE), a kind of covalent protein complex, in analogy to advanced glycation end products (AGE) (Farmer and Davoine, 2007). Furthermore, the production of MDA also indicated the level of oxidative stress in an organism (Moore and Roberts, 1998; Del Rio et al., 2005). In this research, high levels of MDA were measured after NEFA treatment for 3 h.

Glutathione is an endogenous antioxidant that protects tissues from oxidative stress by reducing hydroperoxides and organic peroxides, including lipid peroxide, protecting the cell membrane from free radical oxidative damage during redox reactions. GSH is considered to be a free radical scavenger of ROS and an inhibitor of lipid peroxidation (Owen and Butterfield, 2010). The redox function in cells protects the cell membrane from free radical oxidation damage (Zitka et al., 2012). In this research, low levels of GSH after NEFA treatment for 1 h indicated an increase in oxidative stress.

As is well-known, the pathogenic process of NAFLD is linked to aberrant lipid accumulation, insulin resistance, and inflammation in hepatocytes. Previous reports have shown that a high concentration of NEFAs has been present in human patients (Giacomini et al., 1980), as well as in cattle and animal models with NAFLD. In the present study, we added a high concentration of NEFAs into BRL-3A cell lines for different times in the pathologic process of NAFLD. The results showed that oxidative stress appeared from 1 to 9 h, and ER stress appeared from 6 to 12 h. This finding indicates that NEFAs induced mitochondrial dysfunction and then ER stress.

ROS acts as a major regulator in the maintenance of homeostasis of ER stress involving UPR-mediated cell signaling, leading to UPR activation and restoration of ER homeostasis (Zeeshan et al., 2016). GSH plays a key role in maintaining ER oxidoreductases in a reduced state, and was considered to induce calcium release via inositol triphosphate receptors (IP3R) (Chan et al., 2016; Zeeshan et al., 2016). Enhanced mitochondrial Ca^2+^ increased metabolism activities and ROS generation in mitochondria (Gorlach et al., 2006). In this research, store-operated Ca^2+^ entry increased after NEFA treatment for 0.5 h. Therefore, we speculate that intracellular Ca^2+^ correlated with decreased GSH.

Intracellular Ca^2+^ plays an important role in signal transduction in various cell types, involving cell proliferation, cell death, migration, and metabolism (Lang et al., 2012; Ivanova et al., 2017). Orai1, the moiety of SOCE, was activated by NEFAs and facilitates Ca^2+^ entry to maintain Ca^2+^ balance. In addition, we also added Ca^2+^ inhibitor BTP_2_ and silencing Orai1 into BRL-3A cell lines before NEFA treatment, and the results showed that NEFA could activate mitochondrial ROS production and cause ER stress; conversely, the effects were decreased by inhibitor BTP_2_ and silencing Orai1. Orai1 expression was shown to be up-regulated by NFκB (Lang and Shumilina, 2013). The present study demonstrates that an NFκB inhibitor reduces Orai1 expression. Moreover, oxidative stress and ER stress were also down-regulated by the NFκB inhibitor wogonin. The results also showed that when Orai1 is silenced, the content of p65 translocated to the nucleus could be reduced. This suggests that Orai1 may interact with p65. Thus, we suspect that it may be effective in inflammation after silencing Orai1. The present observations, however, do not rule out that NFκB could play a permissive role in the regulation of oxidative stress and ER stress in the pathologic process of NAFLD. The NFκB–Orai1 pathway plays an important role in the inflammatory responses that regulate hepatic lipid deposition in the pathological process of NAFLD. Inhibition of the NFκB–Orai1 pathway could reduce NEFA-induced oxidative stress, thus decreasing ER stress and causing NAFLD. Taken together, the decrease in GSH, caused by NEFAs induced in earlier stages of oxidative stress, induced calcium release and further enhanced the sensitivity of calcium channels, Orai1. NFκB could be activated after Ca^2+^ entry to enhance Orai1 expression and is thus part of a positive feedback further augmenting Ca^2+^ influx (Eylenstein et al., 2012). Increased levels of mitochondrial Ca^2+^ could increase ROS generation in mitochondria and further enhance ER stress.

## Conclusion

In conclusion, the present study is the first to clearly demonstrate the involvement of NEFA-sensitive NFκB–Orai1 signaling in the regulation of oxidative stress and ER stress in the pathological process of NAFLD; moreover, NFκB–Orai1 influences ROS generation and further enhances ER stress.

## Data Availability Statement

All datasets generated for this study are included in the manuscript/Supplementary Files.

## Ethics Statement

The animal study was reviewed and approved by the animal experiments were conducted according to the Chinese law for the welfare of animal and were approved by Heilongjiang Bayi Agricultural University.

## Author Contributions

BBZ, WY, and CXu designed the project. ML, YZ, HG, and BDZ performed the experiments and analyzed the results. CXi and HZ revised the manuscript. BBZ and ML drafted the manuscript. All authors revised the final version of the manuscript.

## Conflict of Interest

The authors declare that the research was conducted in the absence of any commercial or financial relationships that could be construed as a potential conflict of interest.

## Footnotes

1 https://www.thermofisher.com/document-connect/document-connect.html?url =https%3A%2F%2Fassets.thermofisher.com%2FTFS-Assets%2FLSG%2Fmanuals %2FLipofectamine_2000_Reag_protocol.pdf&title=TGlwb2ZlY3RhbWluZSZyZ Wc7IDIwMDAgUmVhZ2VudCBQcm90b2NvbA==

2 http://www.njjcbio.com/products.asp?id=1532

3 https://www.beyotime.com/product/S0131.htm

## Supplementary Material

FIGURE S1 Phospho-p65 protein expressed in BRL-3A cells. **(A)** Original Western blots showing the protein expression of phosph-p65, NFκB p65, and β-actin in BRL-3A cells incubated without or with NEFA (1.2 mM, 12 h) in the absence or presence of Orai1 inhibitor BTP2; arithmetic means ± SEM (*n* = 9) of the phosph-p65/β-actin ratio in BRL-3A cells incubated without (white bar) or with (black bar) NEFA (1.2 mM, 12 h) in the absence (left bars) or presence (right bars) of Orai1 inhibitor BTP2; arithmetic means ± SEM (*n* = 9) of the NFκB p65/β-actin ratio in BRL-3A cells incubated without (white bar) or with (black bar) NEFA (1.2 mM, 12 h) in the absence (left bars) or presence (right bars) of Orai1 inhibitor BTP2. **(B)** Original Western blots showing the protein expression of phosph-p65, NFκB p65, and β-actin in BRL-3A cells incubated without or with NEFA (1.2 mM, 12 h) in the absence or presence of NFκB p65 inhibitor wogonin; arithmetic means ± SEM (*n* = 9) of the phosph-p65/β-actin ratio in BRL-3A cells incubated without (white bar) or with (black bar) NEFA (1.2 mM, 12 h) in the absence (left bars) or presence (right bars) of NFκB p65 inhibitor wogonin; arithmetic means ± SEM (*n* = 9) of the NFκB p65/β-actin ratio in BRL-3A cells incubated without (white bar) or with (black bar) NEFA (1.2 mM, 12 h) in the absence (left bars) or presence (right bars) of NFκB p65 inhibitor wogonin. **(C)** Original Western blots showing the protein expression of phosph-p65, NFκB p65, and β-actin in BRL-3A cells incubated without or with NEFA (1.2 mM, 12 h) in the absence or presence of siOrai1; arithmetic means ± SEM (*n* = 9) of the phosph-p65/β-actin ratio in BRL-3A cells incubated without (white bar) or with (black bar) NEFA (1.2 mM, 12 h) in the absence (left bars) or presence (right bars) of siOrai1; arithmetic means ± SEM (*n* = 9) of the NFκB p65/β-actin ratio in BRL-3A cells incubated without (white bar) or with (black bar) NEFA (1.2 mM, 12 h) in the absence (left bars) or presence (right bars) of siOrai1. ^∗^*p* < 0.05, ^∗∗^*p* < 0.01, indicate significant difference from control; ^#^*p* < 0.05, ^##^*p* < 0.01, indicate significant difference from NEFAs alone (one-way ANOVA).

FIGURE S2 Effect of high-concentration NEFAs and siOrai1 on phospho-NFκB p65 localization in BRL-3A cells. Original immunofluorescence images demonstrating nuclear staining (blue; left images), phospho-NFκB p65 (red; middle images), and an overlaying of all nuclear staining and phospho-NFκB p65-specific antibody in BRL-3A cells incubated without (upper images) or with (second images) NEFAs (1.2 mM, 3 h), siOrai1 (third images), or siOrai1 + NEFAs (lower images). Scale bar: 25 μm.
