# Corrigendum: NFκB/Orai1 Facilitates Endoplasmic Reticulum Stress by Oxidative Stress in the Pathogenesis of Non-alcoholic Fatty Liver Disease

**DOI:** 10.3389/fcell.2019.00290

**Published:** 2019-11-22

**Authors:** Bingbing Zhang, Ming Li, Ying Zou, Han Guo, Bingdong Zhang, Cheng Xia, Hongyou Zhang, Wei Yang, Chuang Xu

**Affiliations:** ^1^College of Life Science and Technology, Heilongjiang Bayi Agricultural University, Daqing, China; ^2^College of Animal Science and Technology, Heilongjiang Bayi Agricultural University, Daqing, China; ^3^Luquan No.3 Hospital, Shijiazhuang, China

**Keywords:** NAFLD, oxidative stress, ER stress, Orai1, NEFA

In the published article, there was an error in affiliation “1” and “2.” Instead of “College of Life Science and Technology, Heilongjiang Bayi Agricultural University, Xinyang, China” and “College of Animal Science and Technology, Heilongjiang Bayi Agricultural University, Xinyang, China”, it should be “College of Life Science and Technology, Heilongjiang Bayi Agricultural University, Daqing, China” and “College of Animal Science and Technology, Heilongjiang Bayi Agricultural University, Daqing, China,” respectively.

The authors apologize for this error and state that this does not change the scientific conclusions of the article in any way. The original article has been updated.

