# Supplementary figures and images for: NFκB/Orai1 Facilitates Endoplasmic Reticulum Stress by Oxidative Stress in the Pathogenesis of Non-alcoholic Fatty Liver Disease
Source: Front Cell Dev Biol. 2019 Oct 2;7:202. doi: 10.3389/fcell.2019.00202 (PMC6783633; doi:10.3389/fcell.2019.00202)

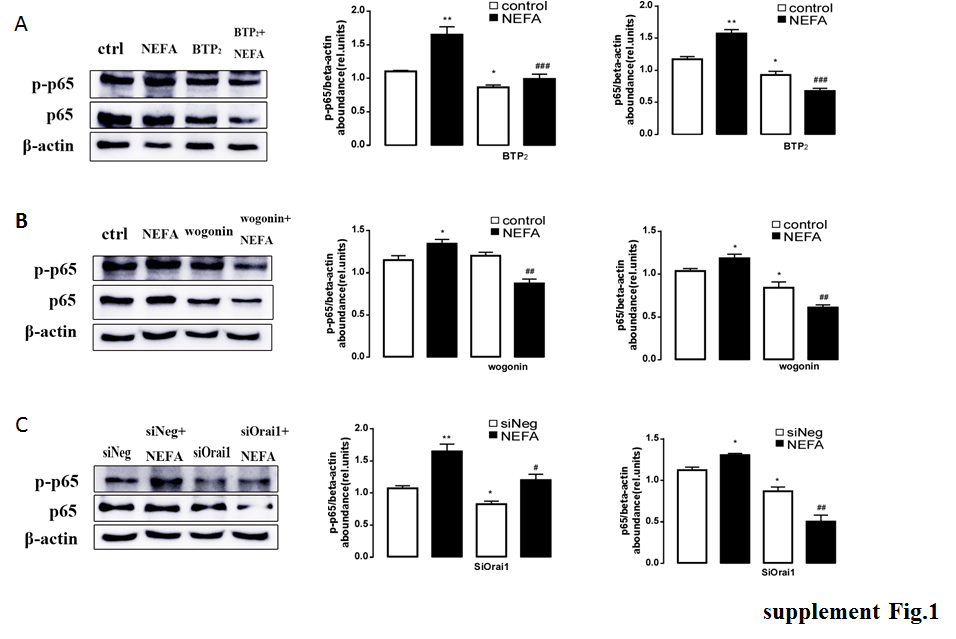

Supplement: FIGURE S1 — Phospho-p65 protein expressed in BRL-3A cells. (A) Original Western blots showing the protein expression of phosph-p65, NFκB p65, and β-actin in BRL-3A cells incubated without or with NEFA (1.2 mM, 12 h) in the absence or presence of Orai1 inhibitor BTP2; arithmetic means ± SEM (n = 9) of the phosph-p65/β-actin ratio in BRL-3A cells incubated without (white bar) or with (black bar) NEFA (1.2 mM, 12 h) in the absence (left bars) or presence (right bars) of Orai1 inhibitor BTP2; arithmetic means ± SEM (n = 9) of the NFκB p65/β-actin ratio in BRL-3A cells incubated without (white bar) or with (black bar) NEFA (1.2 mM, 12 h) in the absence (left bars) or presence (right bars) of Orai1 inhibitor BTP2. (B) Original Western blots showing the protein expression of phosph-p65, NFκB p65, and β-actin in BRL-3A cells incubated without or with NEFA (1.2 mM, 12 h) in the absence or presence of NFκB p65 inhibitor wogonin; arithmetic means ± SEM (n = 9) of the phosph-p65/β-actin ratio in BRL-3A cells incubated without (white bar) or with (black bar) NEFA (1.2 mM, 12 h) in the absence (left bars) or presence (right bars) of NFκB p65 inhibitor wogonin; arithmetic means ± SEM (n = 9) of the NFκB p65/β-actin ratio in BRL-3A cells incubated without (white bar) or with (black bar) NEFA (1.2 mM, 12 h) in the absence (left bars) or presence (right bars) of NFκB p65 inhibitor wogonin. (C) Original Western blots showing the protein expression of phosph-p65, NFκB p65, and β-actin in BRL-3A cells incubated without or with NEFA (1.2 mM, 12 h) in the absence or presence of siOrai1; arithmetic means ± SEM (n = 9) of the phosph-p65/β-actin ratio in BRL-3A cells incubated without (white bar) or with (black bar) NEFA (1.2 mM, 12 h) in the absence (left bars) or presence (right bars) of siOrai1; arithmetic means ± SEM (n = 9) of the NFκB p65/β-actin ratio in BRL-3A cells incubated without (white bar) or with (black bar) NEFA (1.2 mM, 12 h) in the absence (left bars) or presence (right bars) of siOrai [file Image_1.TIF]

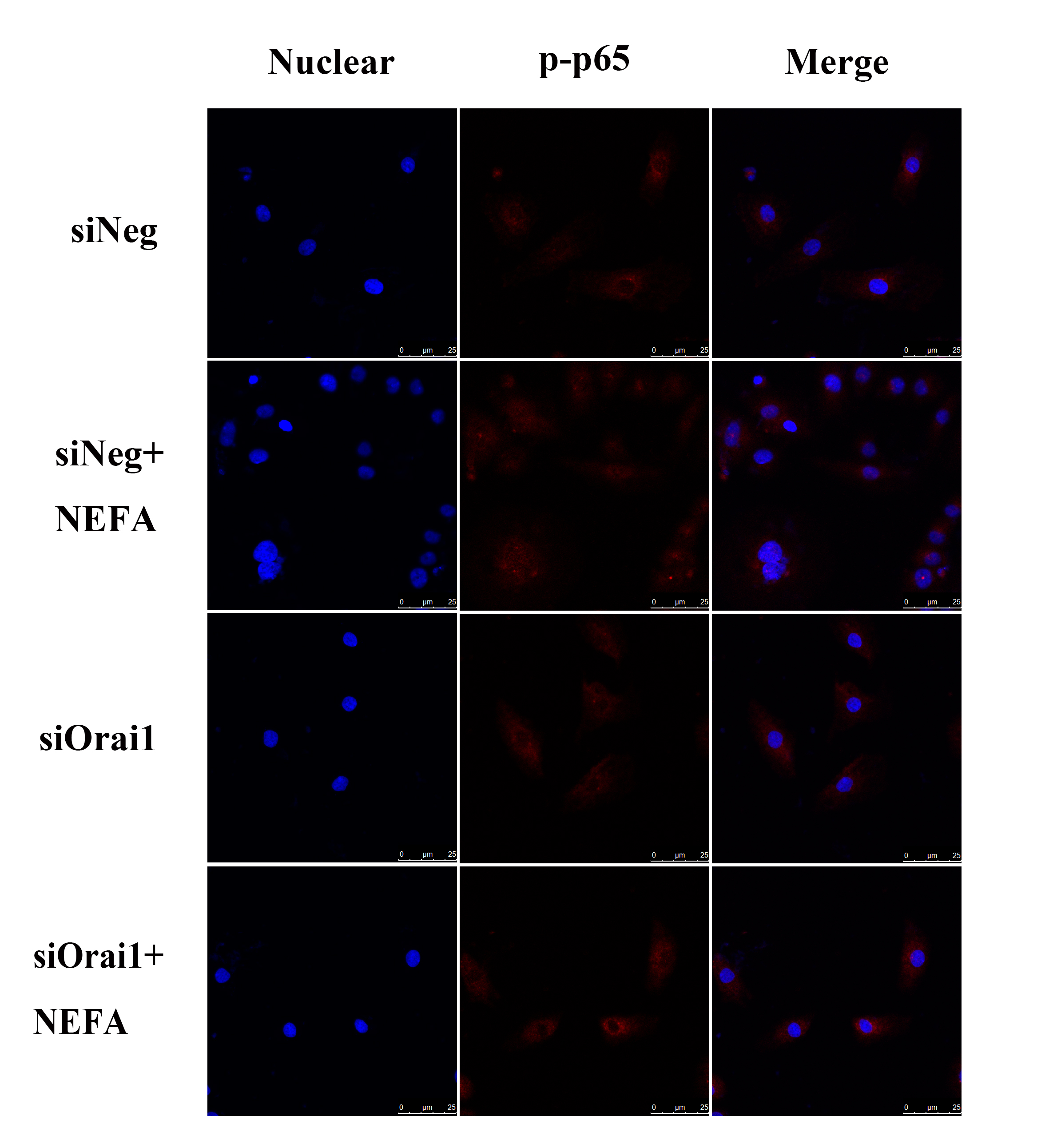

Supplement: FIGURE S2 — Effect of high-concentration NEFAs and siOrai1 on phospho-NFκB p65 localization in BRL-3A cells. Original immunofluorescence images demonstrating nuclear staining (blue; left images), phospho-NFκB p65 (red; middle images), and an overlaying of all nuclear staining and phospho-NFκB p65-specific antibody in BRL-3A cells incubated without (upper images) or with (second images) NEFAs (1.2 mM, 3 h), siOrai1 (third images), or siOrai1 + NEFAs (lower images). Scale bar: 25 μm. [file Image_2.JPEG]
